# Stretching for Recovery from Groin Pain or Injury in Athletes: A Critical and Systematic Review

**DOI:** 10.3390/jfmk6030073

**Published:** 2021-08-30

**Authors:** José Afonso, João Gustavo Claudino, Hélder Fonseca, Daniel Moreira-Gonçalves, Victor Ferreira, José Marques Almeida, Filipe Manuel Clemente, Rodrigo Ramirez-Campillo

**Affiliations:** 1 Centre for Research, Education, Innovation, and Intervention in Sport (CIFI_2_D), Faculty of Sport of the University of Porto (FADEUP), 4200-450 Porto, Portugal; jneves@fade.up.pt (J.A.); victorferreiraster@gmail.com (V.F.); josealmeida5@gmail.com (J.M.A.); 2 Laboratory of Biomechanics, School of Physical Education and Sport, Universidade de São Paulo, São Paulo 05403-010, Brazil; claudinojgo@usp.br; 3 Research and Development Department, LOAD CONTROL, Contagem 32280-440, Brazil; 4 Research Centre in Physical Activity, Health and Leisure, Faculty of Sport of the University of Porto (FADEUP), 4200-450 Porto, Portugal; hfonseca@fade.up.pt (H.F.); danielmgon@fade.up.pt (D.M.-G.); 5 Laboratory for Integrative and Translational Research in Population Health (ITR), 4050-091 Porto, Portugal; 6 Escola Superior Desporto e Lazer, Instituto Politécnico de Viana do Castelo, Rua Escola Industrial e Comercial de Nun’Álvares, 4900-347 Viana do Castelo, Portugal; 7 Instituto de Telecomunicações, Delegação da Covilhã, 1049-001 Lisboa, Portugal; 8 Department of Physical Activity Sciences, Universidad de Los Lagos, Santiago 8320000, Chile; r.ramirez@ulagos.cl; 9 Centro de Investigación en Fisiología del Ejercicio, Facultad de Ciencias, Universidad Mayor, Santiago 7500000, Chile

**Keywords:** pubalgia, flexibility, return to sport, musculoskeletal pain, rehabilitation, exercise training

## Abstract

Stretching is usually used as part of rehabilitation protocols for groin pain or injury, but its specific contribution to and within multimodal recovery protocols is unclear. Our goal was to systematically review the effects of stretching for the recovery from groin pain or injury. The Preferred Reporting Items for Systematic Reviews and Meta-Analyses (PRISMA) guidelines were followed, with eligibility criteria defined according to PICOS: (Participants) athletes with groin pain or injuries; (Interventions) interventions with stretching as the differentiating factor; (Comparators) comparators not applying stretching; (Outcomes) symptom remission or improvement and/or time to return to sport and/or return to play; (Study design) randomized controlled trials. Searches were performed on 26 March 2021, in CINAHL, Cochrane Library, EBSCO, EMBASE, PEDro, PubMed, Scielo, Scopus, SPORTDiscus, and Web of Science, with no limitations regarding language or date, and no filters. Of 117 retrieved results, 65 were duplicates and 49 were excluded at the screening stage. The three articles eligible for full-text analysis failed to comply with one or more inclusion criteria (participants, intervention and/or comparators). We then went beyond the protocol and searched for non-randomized trials and case series, but no intervention was found where stretching was the differentiating factor. We found no trials specifically assessing the effects of stretching on recovery or improvement of groin pain or injury in athletes. Currently, the efficacy of these interventions is unknown, and more research is warranted.

## 1. Introduction

Groin pain or injury (GPI) is a common clinical condition in athletes from different sports [1,2,3]. While GPI has been reported in ice hockey [4] and basketball [5], it is particularly well-described in football (i.e., soccer), with injury rates as high as ~111 per 100,000 men footballers at collegiate level [6]. A cross-sectional cohort study with 695 sub-elite male football players showed that ~50% of the athletes reported hip and/or groin pain in the previous season [7], but unfortunately the percentage specific to GPI was not reported. Although the problem is more common in men [8], the prevalence of groin injuries can reach 40% in women footballers [9].

GPI is an umbrella concept that includes distinct pathological processes (potentially overlapping) and multifactorial causes [1,10,11,12], making interpretation of the literature complex. The lack of agreement on definitions and terminology of GPI as well as the various definitions of what constitutes a sports injury [13] challenge our ability to interpret the findings. Moreover, studies addressing the role of therapeutic exercises in the context of GPI are highly heterogeneous in terms of sample (e.g., different sports and age groups), diagnosis (i.e., different underlying causes for groin pain) and protocols (i.e., distinct exercise types, as well as loads) [2,3,11] and, overall, of low quality [3,14]. Likewise, adductor strength, range of motion, and pain to palpation are surrogate outcomes that operate as limited proxies of recovery status [15]. Despite these limitations, there is evidence that strength and coordination exercises targeting the hip, pelvis, and abdomen are effective in recovery or improvement of GPI, resulting in diminished symptoms and reduced time to return to sport [10,11,12,14,16,17]. The Hölmich protocols and their variations are strength-, coordination-, and balance-based interventions that seem promising to resolve long-term GPI in athletes [14,16,17].

However, poor adductor flexibility has been previously associated with groin pain and injury [8]. Stretching has therefore been hypothesized to aid in recovery from GPI [18], despite lacking a clear cause–effect relationship. The proposed mechanisms through which stretching may alleviate GPI symptoms and accelerate recovery include improvements in hip joint range of motion (ROM) [8,18] and reduction in muscle stiffness [8]. Incidentally, strength training may also improve ROM [19,20]. In healthy adults, passive static stretching has been shown to improve systemic and local vascular function [21] and increase pressure pain thresholds [22]. While it is unclear whether these effects could aid in resolving GPI or merely operate as short-term band-aids to alleviate the symptoms, there is rationale to explore its benefits.

Recent systematic reviews showed that stretching is commonly used as an adjunct to multimodal exercise and/or therapy programs prescribed for GPI recovery/improvement [1,8,10,11,12,14]. A randomized study with 48 athletes with GPI showed that an exercise therapy program including stretching provided a faster time to return to full sports participation than an exercise-based program not including stretching (consisting of aerobic, strength, and balance drills) [23], but the authors stated that neither treatment was particularly effective, with only 50–55% of the athletes in both groups making a full return to sports. Still, the isolated effect of stretching on groin pain and injury is poorly understood, as its application seems restricted to being an adjunct in multimodal interventions. Here, we use the term “isolated” to denote that stretching is being used as the differentiating factor between groups and not in the sense that stretching is the only therapeutic intervention. In this broader sense, interventions may still be multimodal, but the effects of stretching can only be assessed if this is the sole differentiating element.

Considering that the specific effects of stretching in recovery of GPI are not well known, allied to the lack of consensus in existing research and reduced statistical power of some individual studies [24,25], a systematic review may provide a summary of evidence and promote better informed clinical practice [26,27]. Recent, high-quality reviews on recovery from GPI are available [1,8,10,11,12,14], but these were broad-scope works, considering a plethora of interventions, thus reinforcing our criticisms concerning the lack of assessment of unimodal interventions and consequently a poor knowledge of the effectiveness of each specific component of an intervention. As such, these reviews provided very little information on the specific role of stretching and its effectiveness in enhancing the recovery from GPI. Moreover, the gold standard evidence is provided by randomized controlled trials (RCTs) [27,28], and so best-evidence should be largely grounded on RCTs, except where these are not viable [27]. We contend that it is viable to implement two interventions in a randomized manner, where both arms perform similar interventions, but one arm performs stretching in addition to the baseline intervention, and therefore that there is no scientific justification for the lack of high-quality evidence on the therapeutic effect, or lack thereof, of stretching for GPI treatment.

Our goal was to systematically review the existing RCTs (cluster, parallel, or crossover) where stretching was the sole differentiating intervention factor between the groups, in terms of their effects in symptom remission, return to sport, and return to play.

## 2. Materials and Methods

### 2.1. Protocol and Registration

The Preferred Reporting Items for Systematic Reviews and Meta-Analyses Protocols (PRISMA) guidelines were followed [29,30], as well as the AMSTAR 2 recommendations [31], to assist in structuring this systematic review. Where possible, we updated the structure to better conform to the recently updated PRISMA 2020 guidelines [32]. We planned to use The Grading of Recommendations Assessment, Development, and Evaluation (GRADE) to assess the quality of studies [33].

### 2.2. Eligibility Criteria

Articles were eligible if published in peer-reviewed journals, regardless of language or date [27]. Definitions of Participants, Interventions, Comparators, Outcomes, and Study Design (PICOS) are established in Table 1. Following the pre-established rationale, only RCTs were included to reduce risk of bias, to balance participants across groups, and avoid systematic differences between the groups in terms of confounders [27,34,35]. Included RCTs could use a cluster, parallel, or crossover design [36,37].

### 2.3. Information Sources

The following databases were used to search and retrieve the articles on 26 March 2021: CINAHL, Cochrane Library, EBSCO, EMBASE, PEDro, PubMed, Scielo, Scopus, SPORTDiscus, and Web of Science (all databases/collections).

### 2.4. Search Strategy

Using Boolean operators, the title, abstracts, or keywords had to include: (“groin pain” OR “groin strain” OR “groin injur*” OR “pubalgia” OR “symphysis syndrome” OR “osteitis pubis” OR “adductor tendin*” OR “incipient hernia” OR “inguinal hernia”) AND (“stretch *” OR “mobility” OR “range of motion” OR “flexi *”) AND (“player” OR “athlete *” OR “practitioner” OR “sport *” OR “training” OR “exercise” OR “rehabilitation”) AND (random *). Free text terms were used as they are broader than MeSH terms and do not rely on indexation (as delays in indexation would result in very recent works not emerging in the searches). Furthermore, most databases do not afford searching with MeSH terms.

Specificities of each database: (i) in CINAHL, EBSCO, EMBASE, Scielo, and SPORTDiscus, search was open to all fields; (ii) in PEDro, search was conducted using only the first two lines of code with the option to match any search term OR one code line in abstract and title, the other in title only, and then the order was reversed; as no results were provided, we then attempted to use only the first line of code in title and abstract, matching any search term; (iii) in Web of Science, the combination of title, abstract, and keywords was designated “topic”. No filters were applied.

Specific example from PubMed, on 26 March 2021:

(((“groin pain” [Title/Abstract] OR “groin strain” [Title/Abstract] OR “groin injur *” [Title/Abstract] OR “pubalgia” [Title/Abstract] OR “symphysis syndrome” [Title/Abstract] OR “osteitis pubis” [Title/Abstract] OR “adductor tendin *” [Title/Abstract] OR “incipient hernia” [Title/Abstract] OR “inguinal hernia” [Title/Abstract]) AND (“stretch *” [Title/Abstract] OR “mobility” [Title/Abstract] OR “range of motion” [Title/Abstract] OR “flexi *” [Title/Abstract])) AND (“player” [Title/Abstract] OR “athlet *” [Title/Abstract] OR “practitioner” [Title/Abstract] OR “sport *” [Title/Abstract] OR “training” [Title/Abstract] OR “exercise” [Title/Abstract] OR “rehabilitation” [Title/Abstract])) AND (random * [Title/Abstract])

### 2.5. Selection Process

Two authors independently performed the search, removal of duplicates, screening of titles and abstracts, and analysis of full texts, while a third author reviewed this process. There were no disagreements to report, with 100% inter-rater agreement.

### 2.6. Data Collection Process and Data Items

Extracted data was defined *a priori* to avoid biased processes [36]. Characteristics of individual studies: (i) characterization of sample (e.g., size, population, gender, training status, sport, geographical location); (ii) diagnosis and duration of symptoms; (iii) characterization of interventions and comparators (e.g., length, weekly frequency, modality of stretching or comparator, description of training volume and intensity, number of exercises, description of co-interventions, existence of supervision, supervision ratio, and qualification of supervisors); (iv) adherence rates; (v) funding and conflicts of interest. Additional information was planned for extraction in the case of crossover trials: length of wash-in and wash-out periods, and carryover effects [36].

Primary outcomes were symptom remission, return to sport, and return to play. Secondary outcomes were recurrence of symptoms, perceived quality of life, functional assessments, strength levels, range of motion, and adverse effects from the interventions. For all outcomes, tools and metrics were described [27]. In the absence of significant carryover effects, results from crossover trials were combined with results from parallel trials [37]. JA and JMA independently extracted the relevant qualitative and quantitative data, while VF reviewed the process.

### 2.7. Study Risk of Bias Assessment

The Cochrane risk-of-bias tool for randomized trials (RoB 2) [38] was applied to assess the retrieved articles. All five dimensions of RoB were assessed: (i) randomization process; (ii) deviations from intended interventions; (iii) missing outcome data; (iv) measurement of the outcome; (v) and selection of the reported results. JA assessed RoB for all studies, while VF independently verified the assessments. Consensus had to be achieved.

### 2.8. Effect Measures and Synthesis Methods

The literature admits utilization of two studies to proceed for a meta-analysis [39]; however, following more robust practices [40,41], and to avoid the risk of having small sample sizes [42,43], a minimum of three studies providing pre- and post-intervention data for the same outcome was established *a priori*. For continuous variables (e.g., return to play, return to sport), data for meta-analysis was extracted in the form of means and standard deviations (SDs), converted to Hedge’s *g* effect sizes (ES) [40,41]. When original articles presented 95% confidence intervals (CIs) or standard error of mean (SEM), means and SDs were extracted using Cochrane’s RevMan Calculator for Microsoft Excel [44]. For dichotomic variables (e.g., symptom remission), risk ratios (RRs) were used, as they are better suited for straightforward clinical interpretation [27]. In case the original studies provided data on the form of odds ratios (ORs) or hazard ratios (HRs), these were first converted into RRs using Cochrane proposed formulas [27]. The risk was considered with respect to the event occurring, i.e., remission being established. If no event was observed in one or more arms of a study, a standardized value of 0.5 was added. When no event was observed in any of the arms, the study was excluded from meta-analysis regarding remission. An intention-to-treat analysis was considered for all cases [27].

To guarantee that trials had a proportionate weight depending on the size of their standard errors, and to account for the across-studies heterogeneity, an inverse variance random-effects model was applied [45,46]. All ESs were accompanied by 95% CIs, and their interpretation relied on thresholds established by Hopkins et al. [47]: <0.2, trivial; 0.2–0.6, small; >0.6–1.2, moderate; >1.2–2.0, large; >2.0–4.0, very large; >4.0, extremely large. The I^2^ statistic was applied to determine heterogeneity, and interpretation relied on the following thresholds: low (<25%), moderate (25–75%) and high (>75%) [48].

Moderated analyses were planned to use a random-effects model and independently calculated single factor analysis. When possible, the median split technique was planned [49,50]. Planned subgroup analysis: (i) RoB in randomization process; (ii) RoB in measurement of the outcome; (iii) sex of the participants; (iv) length and weekly frequency of the interventions; (v) stretching modality (i.e., static, dynamic, proprioceptive neuromuscular facilitation, other); (vi) type of diagnostic (e.g., specific diagnosis versus idiopathic); and (vii) supervised vs. unsupervised interventions.

Meta-regression was planned in case a minimum of 10 articles had relevant data for any given covariable [27]. A multivariate random-effects model was planned for relevant intervention variables (e.g., stretching modality, length of intervention, weekly training frequency). For RRs concerning symptom remission, the log-transformed value of the intervention was used [27].

The Comprehensive Meta-Analysis software (version 2, Biostat, Englewood, NJ, USA) was used to perform meta-analytical comparisons. Significance level was established at *p* ≤ 0.05.

### 2.9. Reporting Bias Assessment

The extended Egger’s test was applied to assess publication bias [51]. When bias was present, the trim and fill method was applied [52], in which case L0 was assumed as the default estimator for missing studies [53].

### 2.10. Certainty Assessment

Using the GRADE framework, RCTs were initially graded as high quality, potentially downgraded on the basis of five dimensions [54]. Risk of bias, inconsistency (i.e., heterogeneity), and publication bias were addressed above. Indirectness and imprecision (through 95% CIs) were also assessed [55,56]. JA, DMG, and HF independently assessed overall quality of the studies and confidence in evidence.

## 3. Results

**Figure 1:**
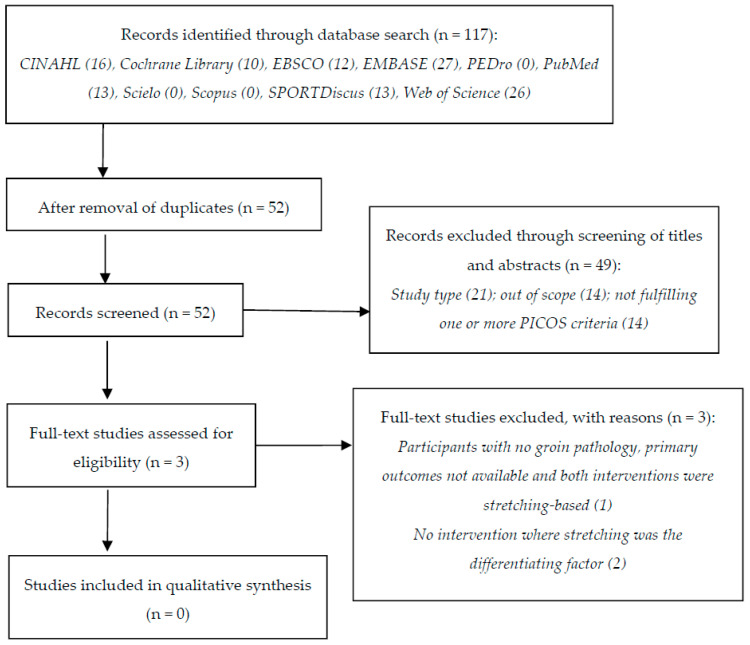
Flowchart describing the study selection process.

The automated search returned 117 results (CINAHL: 16; Cochrane Library: 10; EBSCO: 12; EMBASE: 27; PEDro: 0; PubMed: 13; Scielo: 0; Scopus: 0; SPORTDiscus: 13; Web of Science: 26), reduced to 52 after removal of duplicates. Screening of title and abstract resulted in the exclusion of 49 records: 21 did not fit the type of study (e.g., reviews, essays), 14 were out of scope, and 14 failed to comply with one or more PICOS criteria. Three studies were eligible for full-text analysis [23,57,58], all of which were excluded due to the following reasons: (i) in one study, the participants did not have groin pain, and the primary outcomes defined in our review were not available; further, both intervention and comparator were stretching-based [57]; (ii) in two studies, stretching was not the only differentiating factor between the groups [23,58]. Figure 1 synthesizes the study selection, reflecting an empty review.

## 4. Discussion

### 4.1. Summary of Evidence

Groin pain or injury is an umbrella concept of multifactorial origin [1,11], and represents a common problem in sports [8,9]. Since poor flexibility has been associated with GPI [8], stretching is commonly included in multimodal exercise programs prescribed in this context [1,11,18]. Still, multimodal interventions are not *per se* more effective than unimodal interventions [59], and so it is important to scrutinize the efficacy of each component of an exercise program to better understand its “isolated” effect [60]. If a unimodal program is as effective as multimodal programs, then more focused and time-saving interventions can be delivered [61], potentially increasing adherence to the program and reducing the personal and economic burden of treatment and potential harms [62]. Conversely, if a unimodal intervention is ineffective, adding that intervention to a more comprehensive program will result in time wasted. While several recent, high-quality systematic reviews were published on the topic of recovery from GPI [1,8,10,11,12,14], they have provided reduced information concerning the specific benefits of stretching in this context.

Therefore, our goal was to systematically review the effects of randomized stretching interventions on recovery or improvement of GPI in athletes. The interventions had to be stretching-based; however, as specified in the methods, athletes could engage in regular sports training. Despite the clinical narrative supporting the use of stretching for the recovery or improvement of GPI [3,8,18], gold-standard evidence derives from RCTs [27,28,34] where stretching is the differentiating factor between intervention groups.

Surprisingly, from the ten (relevant) electronic databases searched (with no filters or limitations such as language or publication date), no eligible studies were found meeting the inclusion criteria defined in our pre-registered protocol. This means that there is a lack of gold-standard evidence supporting the utilization of stretching for athletes with GPI. This is somewhat surprising, considering that it is feasible to implement RCTs under real-life conditions: the athletes can perform their regular training sessions and therapies, with stretching being used as the differentiating factor. For example, one study (although not focused on GPI, and therefore not included in our review) analyzed sixteen dancers with hamstring injuries that were randomly assigned to a stretching group, with another group receiving conventional treatment (analgesics and physical therapy) [63]. In the case of athletes with GPI, another RCT assessed the effects of wearing compression garments versus not wearing them, with all other factors being equal [64]: this article had no stretching intervention, and so was not included in our review. There is no apparent reason for not conducting such trials where stretching is the differentiating factor.

While we searched ten databases and did not restrict language or date, the limitation of study design to RCTs may have detracted from finding other useful information related to our research question. At this point, we broke protocol and searched for investigations using alternative study designs.

### 4.2. Evidence from Other Study Designs

The purpose of a pre-registered protocol is to provide a transparent account of the scientific process, and, especially, to avoiding selective reporting of outcomes and analyses [65]. We followed the pre-registered protocol, but here we felt the need to incorporate information from non-randomized study designs. This process was performed *a posteriori*, based on the inexistence of RCTs fulfilling our eligibility criteria.

A case series of six athletes with groin pain (4 males, 2 females; 19–22 years of age) adopted a physical therapy algorithm that included passive stretching and proprioceptive neuromuscular facilitation [18]. Three athletes returned to sport after a mean of 7.7 physical therapy sessions, while the other three required surgical repair, followed by a mean of 6.7 physical therapy sessions. The therapy also included soft tissue mobilization techniques (e.g., effleurage, petrissage) and isometric strength training, and so it is unclear what contribution of stretching had in the overall recovery.

A case report of a 20-years-old male Australian Rules football player with groin pain described a resolution of signs and symptoms after four weeks of a multimodal therapy, including spinal manipulative therapy, myofascial release, and proprioceptive neuromuscular facilitation stretching, among other techniques [66], again making it impossible to assess the specific contributions of stretching to the recovery.

Furthermore, while case series stimulate the generation of hypotheses and are interesting for detecting novelties, their findings should not be generalized, and they are unable to establish causal relationships [67]. Without control or comparative groups, there is nothing against which to compare the outcomes [68,69]. Evidence-based medicine therefore requires a careful selection of controls, i.e., subjects that will have similar characteristics to the treatment group, differing only in the intervention of interest [70]. Within this context, the aforementioned randomized study in athletes with GPI compared six weeks of exercise therapy (consisting of isometric and dynamic strength training, balance training, aerobic training, sprinting, and change of direction training) to a multimodal therapy consisting of heat, manual therapy, and stretching [23]. Despite being randomized, stretching was not the single differentiating factor between the interventions.

In summary, protocols for groin pain are not implementing stretching as the differentiating factor, regardless of study design, and so the effects of stretching on the recovery or improvement of GPI is currently unclear. This also illustrates that a less stringent definition of study design in the eligibility criteria would not have changed the study conclusions.

### 4.3. The Implications of an Empty Review for the Field

Our research resulted in an empty review [71,72]. Globally, it is important for science that all data is published, to promote a more balanced account of the phenomena being studied [73,74,75]. Specifically, empty reviews are relevant and useful for the advancement of evidence-based practices, highlighting major research gaps [71,72,76,77]. For example, long-standing traditions (e.g., clinical practices; position stands) may be exposed as not being sustained by evidence, and this may justify funding for performing future research on the topic [28,72,78]. This knowledge may also influence whether clinicians and patients will apply a certain intervention [28,78]. In the current case, comparative interventions where stretching is applied to one arm of the intervention, but not to the other arms, are needed.

Occasionally, researchers are aware that few RCTs may exist for a specific subject, but it is only after performing a systematic review that more definitive statements can be elaborated [28,79]. Although alternative formats (e.g., narrative reviews) represent interesting alternatives, the lack of a systematic search and selection strategy can easily bias the reporting and analysis of the existing body of knowledge. Indeed, even clinical practice guidelines have been criticized for the lack of a systematic approach and consequent vulnerability to several biases [80]. While narrative reviews may be great for educational purposes, asking broad research questions and advancing the conceptual organization and understanding of a field of research, systematic reviews are better suited for synthesizing data concerning a narrower research question while reducing the risk of bias [65,81]. Our narrow clinical question, allied to our intention of providing reproducible findings, resulted in choosing the format of a systematic review [79].

Unfortunately, empty systematic reviews remain under-reported, being vulnerable to publication bias and difficult to publish in academic journals [71,77]. Consequently, unsubstantiated opinions or pseudoscience may gain popularity and pass as scientifically sustained concepts [76,82]. However, in science, the burden of proof falls on the shoulders of the proponents [83,84,85]. Still, several practical proposals may be put forth and implemented without a corresponding evidence-based assessment [86].

Still, empty reviews have been published in several fields, including autism spectrum disorder [76], diabetic neuropathy [87], educational strategies in the context of frailty prevention and management [86], nursing [88], and tactical periodization in sport [82]. Until August of 2010, empty reviews comprised 8.7% of active reviews in the Cochrane Database of Systematic Reviews, and 45 of the 53 Review Groups had at least one empty review [72]. In our review, there was no evidence from RCTs of an effect of stretching (when compared to passive or active control condition) on recovery or improvement of GPI in athletes. Furthermore, when we broke protocol and provided an *a posteriori* account of non-randomized studies, the same conclusion was achieved. While “no evidence of an effect” does not mean “evidence of no effect”, we contend that the burden of proof should apply [82,83,85]. Furthermore, stretching for management of GPI is not a new concept [89,90], and so there has been enough time to properly investigate its effects.

Although the absence of gold-standard research (i.e., RCTs) on the topic should not paralyze clinical or coaching decision-making, strong opinions on whether stretching is effective in this context should be withheld until more solid evidence is provided. Future studies should randomize athletes with GPI into groups where the differentiating factor is stretching, which is mostly feasible and safe for all the groups.

### 4.4. Limitations and Strengths

Restriction to RCTs may be viewed as a limitation, but as previously justified, the goal of our systematic review was to assess only gold-standard evidence, which emerges from RCTs [27,28,34]. Indeed, reviews should include non-randomized studies, mostly to analyze rare effects that would unlikely be assessed using randomized trials [27]; however, GPI is very common in athletes and stretching is usually applied embedded in multimodal interventions. Furthermore, no restrictions were placed concerning language or publication date, and ten relevant databases were searched, with no filters applied. In six of the databases, due to specificities of their search engines, searches followed a more open process (e.g., all fields instead of just title or abstract). Additionally, many potential search terms were used, to avoid narrowing the search excessively, and, in Section 4.2 of the discussion, we considered evidence emerging from non-randomized designs, but no support for stretching emerged.

Considering the lack of studies fulfilling eligibility criteria, it could be suggested that a scoping or narrative review was performed, instead of a systematic review. However, scoping reviews do not follow the systematized approaches and the rigor of systematic reviews, and could provide more biased conclusions [88,91]. Systematic approaches should be implemented not only in reviews, but also in elaborating clinical practice guidelines [80]. Finally, we chose not to change the inclusion and exclusion criteria established in the protocol, for two reasons: (i) it would defeat the purpose of having a pre-registered protocol; and (ii) it would change the goals of our review [31,32,65,91]. The inclusion of an analysis of non-randomized trials was decided *a posteriori* and was clearly identified as such.

Finally, lack of evidence is still evidence, and points towards unexplored areas of research, providing important clues for guiding future investigations. The fact that the lack of evidence emerged from a systematic review (and not from a narrative review) strengthens its conclusions and recommendations.

## 5. Conclusions

There are no RCTs assessing the differentiating effects of stretching on recovery or improvement of groin pain or injury in athletes. We also found no support from non-randomized trials. Therefore, currently, the efficacy of these interventions is unknown, and more research is warranted.

## 6. Other Information

Methods and registration of protocol were performed before initiating the systematic review. PROSPERO CRD42021231386.

## Figures and Tables

**Table 1:** Inclusion and exclusion criteria based on scope and PICOS.

Rule	Inclusion Criteria	Exclusion Criteria
**Article type**	Original research in peer-reviewed journals. No constraints regarding language and publication date.	Conference abstracts, books and book chapters, book reviews, editorials, letters to the editor, feasibility and pilot studies, trial registrations, reviews, essays, original research in non-peer-reviewed journals.
**Participants**	Athletes of any age, sex, and training status, diagnosed with GPI (or any equivalent term).	Athletes not diagnosed with GPI. Non-athletes. Non-human animals (e.g., experimental animal models).
**Interventions**	Stretching (e.g., static passive, static active, dynamic, proprioceptive neuromuscular facilitation, other). *	Interventions without stretching. Multimodal interventions (e.g., stretching combined with strength training). †
**Comparators**	Non-exercise controls under conservative care. Controls performing alternative exercise protocols (e.g., balance, strength-training). Multimodal programs including stretching.	Absence of comparators.
**Outcomes**	*Primary outcomes* Symptom remission (e.g., pain, discomfort, functional limitations); return to sport; return to play. *Secondary outcomes (optional)* Recurrence of symptoms. Perceived quality of life, functional assessments, strength levels, range of motion. Adverse effects arising from the interventions.	Absence of the pre-defined primary outcomes.
**Study design**	Randomized controlled trials (cluster, parallel or crossover), with no limitation regarding timeframe for follow-up.	Non-randomized studies. Case reports, case series, observational studies and similar designs.

* Athletes will likely engage in regular sports training. If stretching was the differentiating factor of the intervention in relation to comparators, it was accepted for our purposes. † Although Tai Chi, Pilates, Yoga, and similar interventions have a stretching component, they have additional components focusing on strength and balance. Therefore, they were considered multimodal activities (unless the authors of the studies reported otherwise). GPI—Groin Pain or Injury.

## Data Availability

As no study was eligible for inclusion, there is no data to report beyond what was presented in the manuscript.
